# The effect of music on breastfeeding: a systematic review and meta-analysis of breast milk production, composition, and maternal stress

**DOI:** 10.1186/s13006-026-00867-0

**Published:** 2026-06-23

**Authors:** Shalin Wan Fei Lee, Li Sze Chai, Saloma Pawi

**Affiliations:** Department of Nursing, Faculty of Medicine and Health Sciences, Universiti Malaysia Sarawak (UNIMAS), Kota Samarahan, Sarawak 94300 Malaysia

**Keywords:** Breastfeeding, Music therapy, Auditory stimulation, Lactation, Breast milk volume, Cortisol, Postpartum stress, Systematic review, Meta-analysis

## Abstract

**Background:**

Breastfeeding is the gold standard for infant nutrition, yet maternal stress and anxiety, especially in the Neonatal Intensive Care Unit (NICU) or due to postnatal pain, can inhibit the oxytocin-mediated milk ejection reflex, leading to reduced milk production and early cessation. Music intervention has been proposed as a non-pharmacological strategy to mitigate these stress responses.

**Objective:**

This systematic review and meta-analysis aims to evaluate the effect of music interventions on objective breastfeeding outcomes (milk volume and composition), salivary cortisol stress marker, and psychological well-being in breastfeeding mothers. Unlike previous studies, this review and analysis includes both term and preterm infants across diverse clinical settings and examines the impact of music on physiological (breast milk volume and composition, breastfeeding duration) and psychological outcomes (maternal stress and anxiety levels).

**Methods:**

A comprehensive search of Scopus, PubMed, CINAHL, and Web of Science was conducted for Randomized Controlled Trials (RCTs) published up to 2024. Following PRISMA 2020 guidelines, studies were selected based on their investigation of the effects of music on breastfeeding mothers. The methodological quality of the included studies was assessed using the Cochrane Risk of Bias tool and GRADE framework for certainty of evidence. Data were synthesized both narratively and through meta-analysis where applicable.

**Results:**

Six RCTs with a total of 696 participants met the inclusion criteria. Quality assessment confirmed that all trials somewhat adhered to protocols with “Some Concerns”. Meta-analysis indicated that music significantly increased breast milk volume both immediately and cumulatively (*Z* > 1.96, *p* < 0.0001) with low heterogeneity (I² = 4%). Additionally, music improved breast milk fat content and reduced maternal salivary cortisol stress levels (*Z* > 1.96, *p* < 0.00001) with negligible heterogeneity (I² = 0%) respectively. The overall certainty of evidence is rated as “Moderate to Low,” necessitating cautious clinical interpretation. This rating is primarily due to methodological heterogeneity in milk volume measurements, a lack of participant blinding, and the potential risk of publication bias.

**Conclusion:**

As one of the first reviews to concurrently assess both physiological and psychological breastfeeding outcomes, findings suggest music therapy is a promising, non-invasive adjunct to lactation care. By potentially reducing maternal stress, it may improve milk quantity and composition. However, further high-quality, standardized trials are necessary to carefully evaluate its true clinical magnitude and determine its appropriate integration into routine protocols for long-term breastfeeding success.

**Clinical trial number:**

Not applicable.

**Supplementary Information:**

The online version contains supplementary material available at https://doi.org/10.1186/s13006-026-00867-0.

## Background

Breastfeeding is universally recognized as the gold standard in infant nutrition, offering unmatched immunological, nutritional, and developmental benefits. The World Health Organization (WHO) and UNICEF recommend exclusive breastfeeding for the first six months of life, emphasizing its role in reducing the risk of infant mortality and morbidity, particularly from infectious diseases [1]. For mothers, breastfeeding has been associated with a reduced risk of breast and ovarian cancers, type 2 diabetes, and cardiovascular disease [2]. Despite these well-documented advantages, global exclusive breastfeeding rates remain suboptimal, with many mothers discontinuing breastfeeding earlier than recommended due to various physiological and psychosocial barriers [3].

A key determinant of breastfeeding success is maternal psychological well-being. The neuroendocrine let-down reflex, essential for lactation, is sensitive to maternal emotional states, with stress, pain, fatigue, and anxiety triggering the hypothalamic-pituitary-adrenal (HPA) axis and elevating cortisol levels [4]. Elevated cortisol can inhibit oxytocin release, disrupting the milk ejection reflex and contributing to perceived insufficient milk supply, which is a leading cause of early breastfeeding cessation [5]. Therefore, interventions aimed at reducing maternal anxiety and promoting relaxation are critical for supporting lactation.

Non-pharmacological interventions, particularly in the postpartum period, are preferred due to the potential risks of pharmacological agents passing through breast milk to the infant [6]. Among these, music interventions have gained attention as an accessible, cost-effective, and non-invasive approach. Music therapy has been shown to modulate the autonomic nervous system, shifting from sympathetic (fight-or-flight) dominance to parasympathetic (rest-and-digest) activity [6]. Studies suggest that listening to preferred or calming music can reduce heart rate, blood pressure, and cortisol levels, fostering a physiological environment conducive to the oxytocin-mediated let-down reflex and potentially enhancing milk flow and volume [7].

In addition to its physiological benefits, music interventions have shown promise in addressing Postpartum Depression (PPD), a condition affecting approximately 17% of mothers globally [8]. PPD can create a vicious cycle, where depressive symptoms undermine breastfeeding self-efficacy, which in turn exacerbates depression. Music listening has been found to influence neurotransmitters such as dopamine and serotonin, which are essential for mood regulation. Several randomized controlled trials suggest that music therapy can significantly reduce PPD symptoms by providing an emotional outlet, alleviating psychological distress, and fostering maternal-infant bonding [9, 10]. By alleviating PPD, music may indirectly promote longer breastfeeding duration and exclusivity by enhancing maternal mental resilience and coping capacity.

Although individual studies have examined the effects of music on breastfeeding outcomes, ranging from milk volume in mothers of preterm infants to anxiety reduction in primiparous women, the literature remains fragmented. Methodological variations, including differences in intervention protocols (music type, duration, setting) and outcome measures, limit the generalizability of findings. While the impact of music on mothers of preterm infants in Neonatal Intensive Care Units (NICUs) is relatively well-documented, evidence regarding healthy term infants and breastfeeding mothers in home or community settings is less synthesized. There is a clear need to consolidate these findings, linking music interventions not only to physical lactation metrics but also to the psychological well-being, such as relaxation and PPD status, that supports breastfeeding success.

While several studies have explored the effect of music on breastfeeding mothers, most focus on either physical outcomes (milk volume) or the impact on preterm infants in NICU settings. This review is the first to comprehensively address both psychological and physiological outcomes and to evaluate the effects across a broader population of breastfeeding mothers, including those with term infants.

This systematic review and meta-analysis aims to critically synthesize the existing evidence on the effects of music interventions on breastfeeding mothers with term and preterm infants. Specifically, this review and analysis has examined the impact of music on (1) objective breastfeeding metrics (milk volume, breastfeeding duration), (2) breast milk composition (fat and protein content), and (3) psychological outcomes (the maternal stress marker measured through salivary cortisol levels and Perceived Stress Scale (PSS-14), as well as anxiety levels through State-Trait Anxiety Inventory (STAI)). By consolidating these findings, the review and analysis seeks to provide evidence-based recommendations for healthcare professionals on utilizing music as an adjunct in lactation and maternal mental health management.

## Methods

### Study design and protocol

As part of the university’s project with an identification number of F05/CDRG/1829/2019, this systematic review was conducted in accordance with the Preferred Reporting Items for Systematic Reviews and Meta-Analyses (PRISMA) 2020 guidelines [11]. The completed PRISMA 2020 checklist is provided as Supplementary Material. The protocol was designed to identify, select, and critically appraise evidence on the efficacy of music interventions in influencing physiological and psychological outcomes in breastfeeding mothers. A meta-analysis was performed to pool effect sizes and summarize the impact of music interventions on these outcomes.

### Search strategy

A comprehensive search was conducted across four major electronic databases: Scopus, PubMed (MEDLINE), CINAHL (EBSCOhost), and Web of Science. A combination of Medical Subject Headings (MeSH) and free-text keywords was used to search for relevant articles. Boolean operators (AND, OR) were applied to refine the search.

The specific search string used was:

(“music” OR “music therapy” OR “auditory stimulation” OR “music intervention” OR “lullaby”) AND (“breastfeeding” OR “lactation” OR “breast milk” OR “breastmilk” OR “milk ejection” OR “postpartum”).

According to the Cochrane Handbook [12], the two-concept logic is the golden rule for search string to prevent sensitivity violation and irrelevant papers. Therefore, the search string only included Population (breastfeeding mothers) and Intervention (music). Outcome measures were manually screened from titles and abstracts.

The search strategy was deliberately restricted to published, peer-reviewed literature indexed in major electronic databases. Grey literature and clinical trial registries were not searched to ensure that all included studies had undergone rigorous peer review and contained complete, finalized datasets necessary for methodological quality assessment. The search was further limited to studies published in English. No date restrictions were applied to ensure comprehensive coverage of relevant literature. Reference lists of eligible studies and relevant systematic reviews were also hand-searched for additional studies.

### Eligibility criteria

Studies were selected based on the PICO (Population, Intervention, Comparison, Outcome) framework:

- **Population (P)**: Postpartum women who are breastfeeding (including mothers of both term and preterm infants).
- **Intervention (I)**: Any form of music intervention (example: live music, recorded music, music therapy) administered during the postpartum period and specifically during breastfeeding.
- **Comparison (C)**: Standard care (routine postpartum and lactation care) without music, silence.

**Outcomes (O)**:

- **Primary Outcomes**: Objective breastfeeding metrics (milk volume, breastfeeding duration).
- **Secondary Outcomes**: Breast milk composition (fat and protein content).
- **Tertiary Outcomes**: Maternal physiological marker (cortisol levels) and psychological status (anxiety, stress).
- **Study Design**: Randomized Controlled Trials (RCTs) were included. Qualitative studies, case reports, editorials, and conference abstracts were excluded.

### Study selection

Identified records were recruited and duplicates were removed. Two independent reviewers screened titles and abstracts for eligibility. Full-text articles were retrieved and assessed. Should there be any disagreements, they were to be resolved through discussion or by consulting a third reviewer. Fortunately, a full agreement was reached between the two reviewers.

### Data extraction

Data were extracted using a standardized form, which included: author, year, country, study design, sample size, details of the music intervention (type, duration, frequency), control conditions, and key findings related to physiological and psychological outcomes.

### Quality assessment

The methodological quality of included studies was assessed using the Cochrane Risk of Bias 2 tool (RoB 2), which is a revised version 2 of the Cochrane Risk-of-Bias tool for randomized trials. It is the recommended tool to assess the risk of bias in randomized trials included in Cochrane Reviews, which is structured into a fixed set of domains of bias, focussing on different aspects of trial design, conduct, and reporting. A proposed judgement about the risk of bias arising from each domain is generated by an algorithm, based on answers to the signalling questions [13]. In this review, studies were evaluated across five domains: (1) randomization process, (2) deviations from intended interventions, (3) missing outcome data, (4) measurement of outcomes, and (5) selection of reported results. Each study was categorized as having “Low Risk” (high quality), “Some Concerns” (some concerns, but not high risk), or “High Risk” (violations in at least one domain or multiple concerns).

### Quantitative statistical analysis and data synthesis

Due to expected heterogeneity in intervention protocols such as music genre, duration, measurement techniques etcetera, a narrative synthesis was initially conducted. Findings were grouped by outcome categories: primary (breast milk volume, breastfeeding duration), secondary (milk composition), and tertiary (maternal physiological and psychological outcomes).

Heterogeneity across studies was quantified using the I² statistic, which measures the proportion of variability due to heterogeneity rather than chance (0–40%: low, 30–60%: moderate, 50–90%: substantial, 75–100%: considerable) [14]. Forest plots were used to visually inspect for overlapping confidence intervals.

For meta-analysis, quantitative data from the included RCTs were pooled using Review Manager (RevMan) software (Version 5.4). The primary outcome measure was breast milk volume (mL). The secondary outcome included milk composition (fat content), and the tertiary outcome was the maternal physiological stress marker, namely salivary cortisol. Where included trials reported milk volume at multiple time points, the final or post-intervention measurement was used in the pooled analysis. Formal small-study-effect tests and funnel-plot inspections such as Egger’s test were not performed because the number of included trials per outcome (*k* = 2 to 5) is below the threshold of *k* ≥ 10 as recommended by the Cochrane Handbook [12].

## Results

### PRISMA flow diagram

**Fig. 1:**
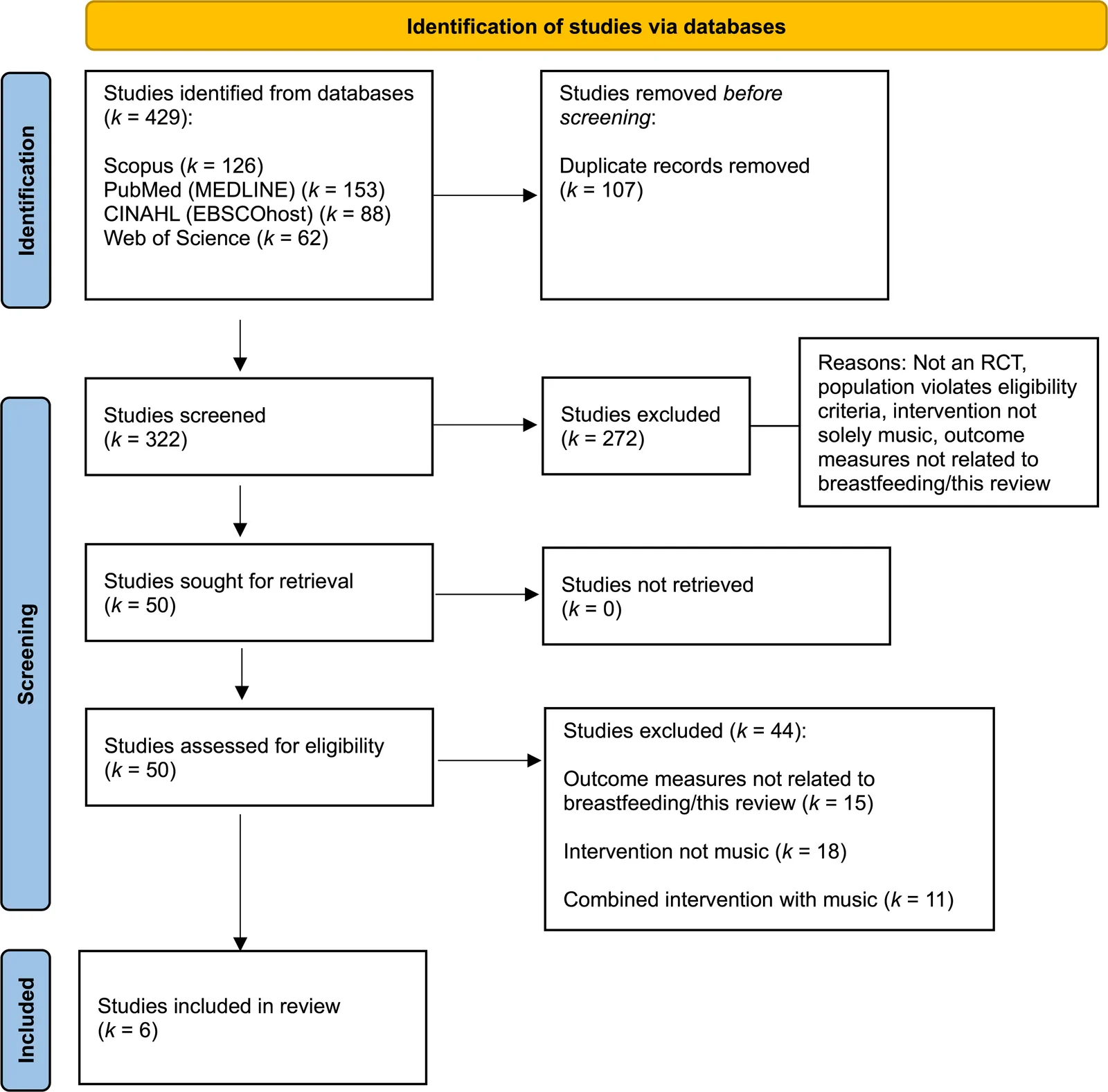
PRISMA 2020 flow diagram

Figure 1 illustrates the flow of information through the phases of the systematic review, following the PRISMA 2020 guidelines [11].

In the identification phase, 429 articles were retrieved from four databases: Scopus (*k* = 126), PubMed (MEDLINE) (*k* = 153), CINAHL (EBSCOhost) (*k* = 88), and Web of Science (*k* = 62). After removing 107 duplicates, 322 studies were screened. Of these, 272 were excluded based on criteria such as non-RCT design, the population is not according to inclusion criteria, non-music interventions, or outcome measures were not related to breastfeeding/this review. Fifty full-text articles were assessed for eligibility, and 44 were excluded due to outcome measures not related to breastfeeding/this review (*k* = 15), non-music interventions (*k* = 18), or combined interventions (*k* = 11). Ultimately, 6 studies were included in the review.

### Data extraction

Table 1 summarizes the key characteristics of the six included studies,which collectively involved 696 participants. These studies were conducted across diverse settings: India, Iran, USA, Thailand, Turkey, and Brazil. Five studies focused on mothers of preterm infants [15, 16, 18–20], while one examined mothers of healthy term infants [17].

Regarding the physiological and psychological outcomes, music interventions were reported to consistently demonstrate positive effects on reducing maternal stress and anxiety [15, 19]. Furthermore, significant improvements in lactation metrics were observed. In particular, several studies [16, 18, 19] reported that music interventions enhanced milk volume and composition. Keith et al. (2012) [16], SefidHaji et al. (2022) [18], and Varişoğlu and Satilmiş (2020) [19] found that mothers of preterm infants who listened to music while pumping produced significantly higher milk volumes and milk with higher fat content compared to control groups. Kittithanesuan et al. (2017) [17] found that, in mothers of healthy term infants, music exposure immediately postpartum led to a significantly shorter time to milk ejection and a higher likelihood of successful lactation initiation (OR = 2.36). Additionally, Vianna et al. (2011) [20] demonstrated long-term benefits, with significantly higher breastfeeding maintenance rates at 15 days post-discharge for mothers exposed to music therapy.

Ak et al. (2015) [15] conducted a study in India involving 30 mothers of premature infants. The study found that a 30-minute session of live raga music (Malkauns and Yaman) played through headphones significantly reduced maternal cortisol levels (*p* = 0.001) and perceived stress (*p* < 0.001). These reductions in stress were associated with a significant increase in breast milk volume compared to the control group (*p* < 0.001).

Kittithanesuan et al. (2017) [17] explored the effects of music on mothers of healthy term infants in Thailand. In this large trial (*n* = 304), those who listened to music immediately after vaginal delivery had a significantly shorter time to milk ejection and a higher likelihood of successful lactation initiation (OR = 2.36) compared to those in the routine care group.

SefidHaji et al. (2022) [18] in Iran compared three groups: standard care, music-only, and music combined with a photo of the infant. While both music groups showed improvements, the combination of music and visual stimuli resulted in the highest milk volume (75.91 mL versus 66.33 mL in controls) and increased milk fat and protein content. However, for this review, the music-only and standard care groups were included in the analysis.

Varişoğlu and Satilmiş (2020) [19] conducted a study in Turkey involving 40 mothers. Although no immediate effects on milk volume were observed on Day 1, significant increases were noted by Day 3 and Day 4. This delayed effect was accompanied by a significant reduction in salivary cortisol levels (*p* < 0.05), indicating that consistent exposure to music over time helped reduce baseline maternal stress and improved lactation performance.

Vianna et al. (2011) [20] in Brazil investigated the long-term impact of music therapy on mothers of hospitalized preterm infants. Their study (*n* = 94) found that regular music therapy sessions significantly increased breastfeeding rates at the first follow-up visit after discharge (*p* = 0.03), suggesting that music therapy may support long-term breastfeeding sustainability.

The small sample sizes in the studies by Ak et al. (2015) [15] and Varişoğlu and Satilmiş (2020) [19], with 30 and 40 participants, respectively, highlight the need for larger-scale studies to increase the statistical power of findings. Furthermore, exploring the combined effect of music and other interventions may provide deeper insights into the factors that influence breastfeeding outcomes.

**Table 1:** data extracted from six selected studies

Author (Year) & Country	Study Design & Sample	Intervention Protocol	Control Group	Outcome Measures	Key Findings
Ak et al. (2015)[15] India	RCT *n* = 30 mothers of premature newborns in NICU.	*n* = 15 30 min of music therapy: 15 min respectively before and during milk expression.	*n* = 15 30 min of milk expression without listening to music.	Breast milk amount (mL). Stress (Perceived Stress Scale / PSS-14). Salivary cortisol assay (nmol/L).	**Primary outcome** ***Milk volume***: Significantly higher milk output in the music group (*p* < 0.001). **Tertiary outcome** ***Psychological (PSS-14)***: Significantly different on day 1 and day 4 (*p* < 0.001). ***Physiological (Salivary cortisol)***: Significantly decrease after music therapy (*p* = 0.001).
Keith et al. (2012)[16] USA	RCT *n* = 162 mothers of preterm infants	*n* = 81 Listening to recorded music (lullabies or classical) for 60 min while pumping milk.	*n* = 81 Standard care (pumping without music).	Milk volume (mL). Fat content (g/dL), caloric content.	**Primary outcome** ***Milk volume***: Mothers in the music group produced significantly more milk compared to controls (*p* < 0.05). **Secondary outcome** ***Milk composition***: Milk fat content was also significantly higher in the music group.
Kittithanesuan et al. (2017) [17] Thailand	RCT *n* = 304 breastfeeding mothers of term infants	*n* = 152 Listening to popular Thai song “Im Oon”) in the delivery room immediately postpartum.	*n* = 152 Routine nursing care without music in the delivery room.	Time to milk ejection. Breast milk volume.	**Primary outcome** ***Milk ejection***: Statistically significant difference in milk ejection between the two groups after suckling (*p* = 0.001). ***Breast milk volume***: The intervention group had higher milk volume compared to the control group (OR = 2.36).
SefidHaji et al. (2022) [18] Iran	RCT *n* = 100 mothers of preterm infants in NICU.	*n* = 33 Group B: Lullaby for 30 min/day for 6 days. *Group C (*n* = 34) not included in review and analysis: Lullaby + viewing baby’s photo for 30 min/day for 6 days.	*n* = 33 Group A: Standard care (no music / photo).	Breast milk volume (mL). Breast milk composition: Fat (Triglycerides, Cholesterol). + Albumin. + Total Protein.	**Primary outcome** ***Mean difference of breast milk volume***: Lullaby group significantly more than control (*p* < 0.001). **Secondary outcome** ***Mean difference of composition***: Lullaby significantly increased milk fat (triglycerides, cholesterol), albumin and total protein levels compared to control (*p* < 0.001) with large effect size of 0.44 and above.
Varişoğlu & Satilmiş (2020)[19] Turkey	RCT *n* = 40 mothers of premature newborns in NICU.	*n* = 20 Listening to music for 15 min during breast milk expression (pumping) sessions, twice daily for 3 days (Days 2–4).	*n* = 20 Standard care: Breast milk expression sessions (15 min) without music.	Breast milk production (mL). State-Trait Anxiety (STAI). Salivary cortisol levels.	**Primary outcome** ***Breast milk production***: Overall, no significant difference in total average milk volume between groups overall. However, milk production was significantly higher in the music group on Day 3 and Day 4 compared to control group. **Tertiary outcome** ***Physiological stress***: Salivary cortisol levels significantly decreased in the music group (*p* < 0.05) but did not change significantly in the control group. ***Anxiety***: Comparisons of pre- and post-STAI scores were significantly lower in the music group compared to controls (*p* = 0.01).
Vianna et al. (2011) [20] Brazil	RCT *n* = 94 mothers of hospitalized preterm infants	*n* = 48 Music therapy sessions (3x/week for 60 min) during hospital stay.	*n* = 46 Usual care.	Breastfeeding rates at discharge and follow-up (7–15 days).	**Primary outcome** ***Breastfeeding rates***: Mothers in the music group had significantly higher breastfeeding rates at the first follow-up visit (*p* = 0.03) compared to the control group.

Note: RCT - Randomized Controlled Trial; STAI - State-Trait Anxiety Inventory

### Methodological quality assessment

The methodological rigor of the six included Randomized Controlled Trials (RCTs) was appraised using the Cochrane Risk of Bias Tool (RoB 2). Implementation of the assessment which focused on five domains: (1) randomization process, (2) deviations from intended interventions, (3) missing outcome data, (4) measurement of outcomes, and (5) selection of reported results, was done through the RoB 2 template and the Excel tool. As summarized in Table 2, while the overall quality of evidence is promising, a common challenge across studies was the inability to blind participants, which is inherent to non-pharmacological interventions.

### Domain 1: randomization process

In the randomization process, sequence generation and allocation concealment are critical for minimizing bias. Most studies demonstrated strong randomization techniques, including computer-generated randomization lists and stratified randomization codes [16, 17]. SefidHaji et al. (2022) [18] and Vianna et al. (2011) [20] employed block randomization, further reducing the risk of selection bias. However, Ak et al. (2015) [15], Varişoğlu and Satilmiş (2020) [19], and Vianna et al. (2011) [20] raised “Some Concerns” due to insufficient detail on allocation concealment, leaving room for potential bias.

### Domain 2: deviations from intended interventions

The most significant risk of bias across all studies arose from deviations in the intervention process, particularly the challenge of blinding. In drug trials, blinding ensures that neither the participant nor the investigator knows the intervention group. However, in music therapy, it is impossible to blind mothers to the intervention. This lack of blinding introduces the risk of performance bias, where the awareness of receiving music therapy could influence self-reported outcomes such as anxiety or stress. Consequently, all studies were rated as having “Some Concerns” in this domain. Two studies [18, 20] explicitly acknowledged this limitation, while some efforts were made to blind data collectors and analysts, reducing observer bias [17, 18, 20].

### Domain 3: attrition and missing outcome data

The rate of attrition and missing data was minimal or well-explained across all studies. Keith et al. (2012) [16] and Vianna et al. (2011) [20] performed intention-to-treat analyses, and no studies reported significant dropout rates. All six studies were rated as “Low Risk” for this domain.

### Domain 4: measurement of outcomes

Objective outcomes, such as milk volume [15–19], biochemical markers [18], and cortisol levels [19], are less susceptible to bias, despite the lack of blinding, and were rated “Low Risk” initially. These outcomes are harder to manipulate through participant beliefs as these were objectively measured.

Nevertheless, there were considerable methodological variations across studies regarding how milk output was measured and how 24-hour production cycles were tracked. Keith et al. (2012) [16] reported acceptable documentation of participants’ daily volume fluctuations although the authors did not specify the brand and type of milk measurement container. In one particular study [17], the endpoint of measuring breast milk volume was at the second hour after birth before mothers were moved out the delivery room. Breast milk production measurements that do not span a full 24-hour period inherently introduced systematic bias, in which the outcomes generally lack high reliability and validity due to significant diurnal variations in feed volumes and high variability between individual nursing sessions [21, 22]. Having said that, measuring the time to milk ejection within the first 2 h after delivery is reliable as a clinical indicator of early lactation success [23], but it is not a valid proxy for total milk volume. The lack of capturing a 24-hour total volume was noted across the other 3 studies [15, 18, 19] as well. The specific details regarding the breast pump models and measuring containers were provided only by Ak et al. (2015) [15] while SefidHaji et al. (2022) [18] explicitly detailed the breast pump model used and all the other studies [16, 17, 19] did neither, raising concerns regarding the accuracy and consistency of the volumetric measurements. Hence, all the 5 studies measuring milk volume were prescribed with “Some Concerns”.

On the other hand, subjective measures like anxiety scores [15, 19] are more vulnerable to detection bias due to the placebo effect from lack of blinding. Thus, these studies were rated “Some Concerns” for subjective outcomes.

### Domain 5: selection of reported results

All six studies appeared to report outcomes consistent with their pre-specified protocols and methodology sections. There were no indications of selective reporting or discrepancies between the planned and reported outcomes, resulting in a “Low” risk of bias in this domain.

**Table 2:** Methodological quality assessment using cochrane risk of bias tool (RoB 2)

Author (Year)	Domain 1: Randomization	Domain 2: Deviations from Interventions	Domain 3: Missing Outcome Data	Domain 4: Measurement of Outcome	Domain 5: Selection of Reported Result	Overall Risk
Ak et al. (2015) [15]	Some Concerns	Some Concerns*	Low	Some Concerns*	Low	Some Concerns
Keith et al. (2012) [16]	Low	Some Concerns*	Low	Some Concerns*	Low	Some Concerns
Kittithanesuan et al. (2017) [17]	Low	Some Concerns*	Low	Some Concerns*	Low	Some Concerns
SefidHaji et al. (2022) [18]	Low	Some Concerns*	Low	Some Concerns*	Low	Some Concerns
Varişoğlu & Satilmiş (2020) [19]	Some Concerns	Some Concerns*	Low	Some Concerns*	Low	Some Concerns
Vianna et al. (2011) [20]	Some Concerns	Some Concerns*	Some Concerns	Low	Low	Some Concerns

Low Risk: Methodology is rigorous and transparent

Some Concerns: Minor issues or lack of detail (e.g., *Participants not blinded, but objective outcomes used and/or assessors blinding implemented)

High Risk: Significant bias potential (e.g., **Participants not blinded AND subjective outcomes used)

### Quantitative synthesis and statistical analysis (meta-analysis)

A total of six Randomized Controlled Trials (RCTs) involving 696 participants were included in the meta-analysis [15–20]. The studies varied in intervention duration (ranging from single sessions to 14 days) and population (mothers of preterm versus term infants), but all utilized auditory stimulation (music) compared to standard care.

**Fig. 2:**
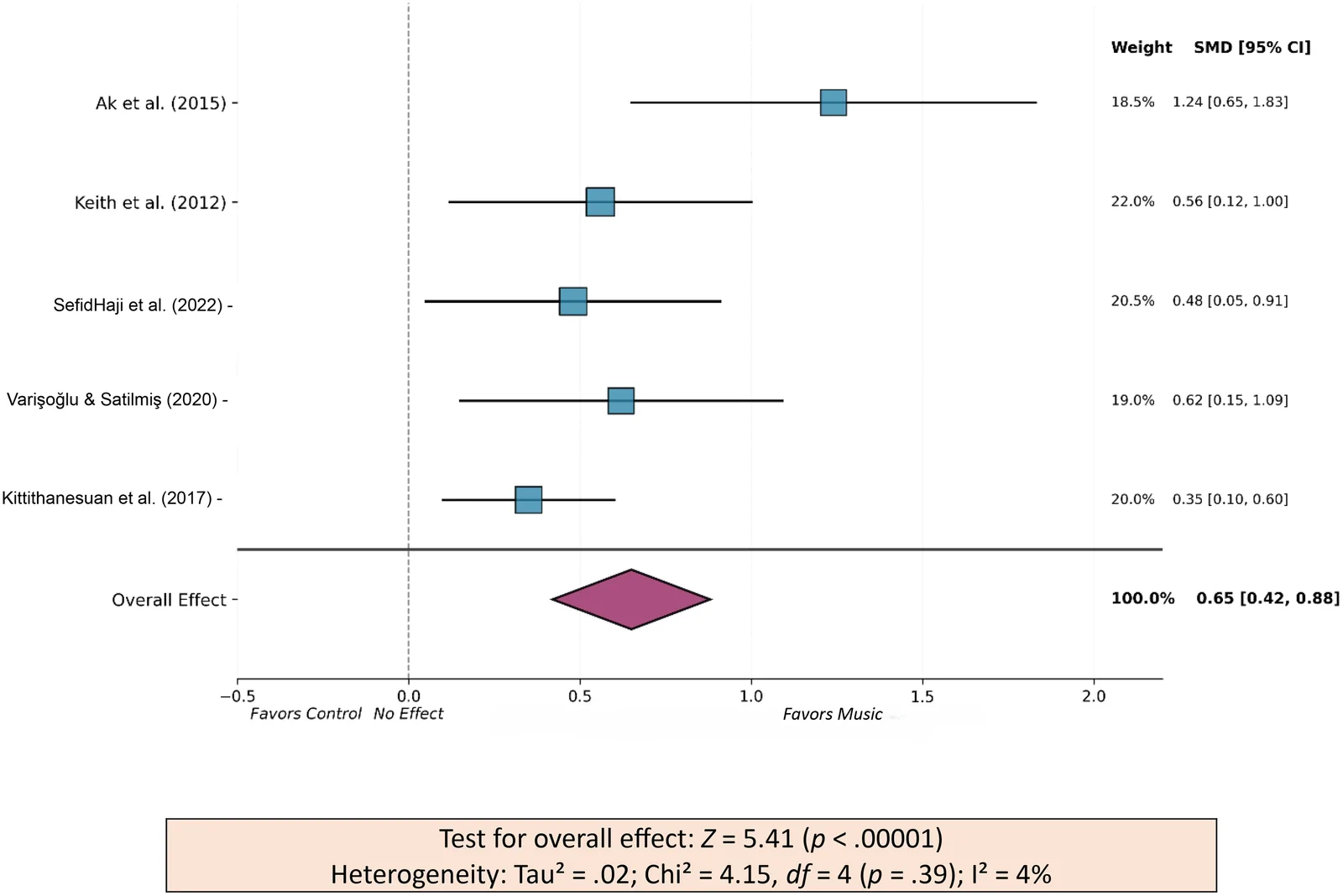
Forest plot – effect of music on breast milk volume

**Fig. 3:**
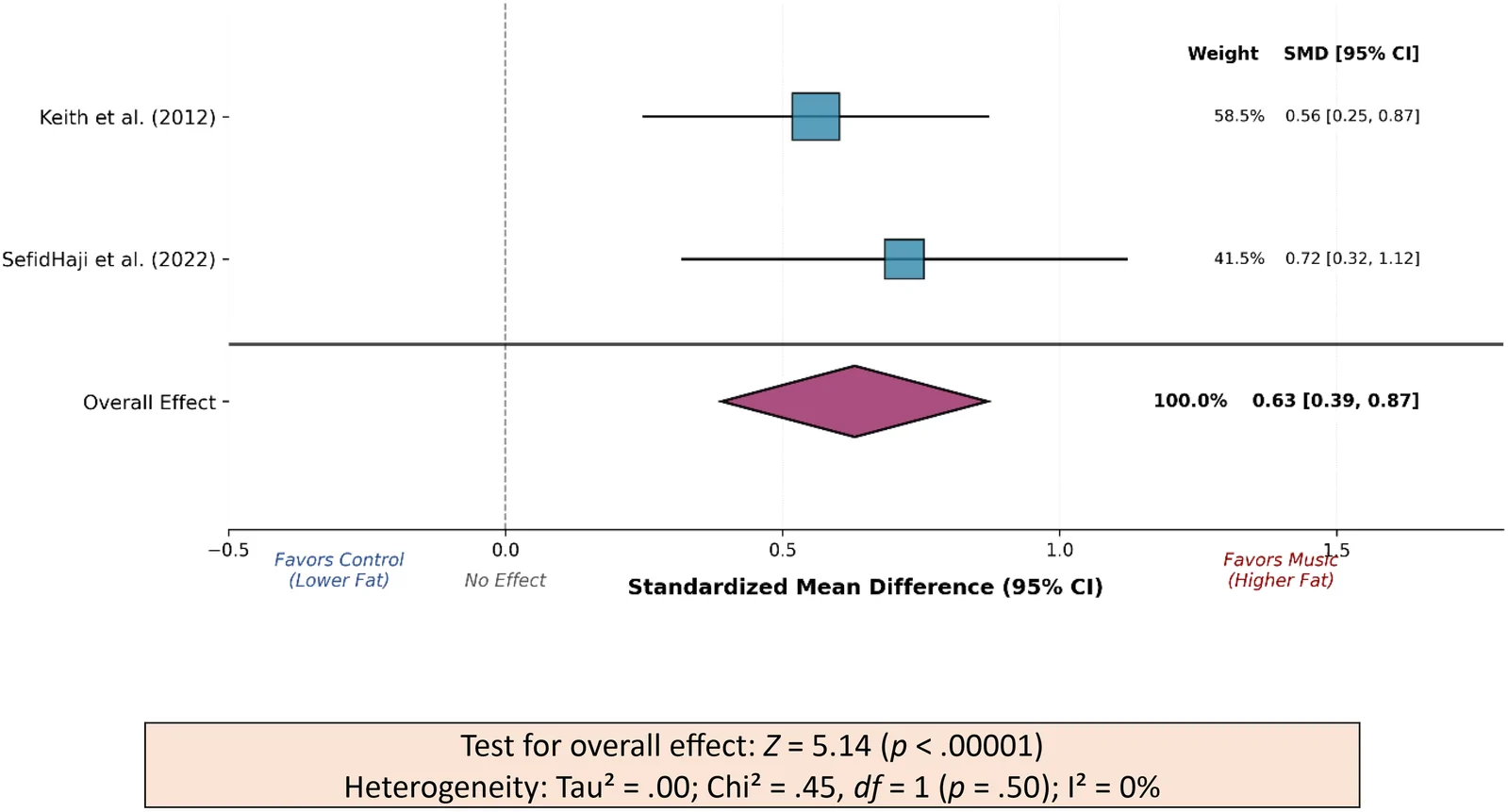
Forest plot – effect of music on breast milk composition.

**Fig. 4:**
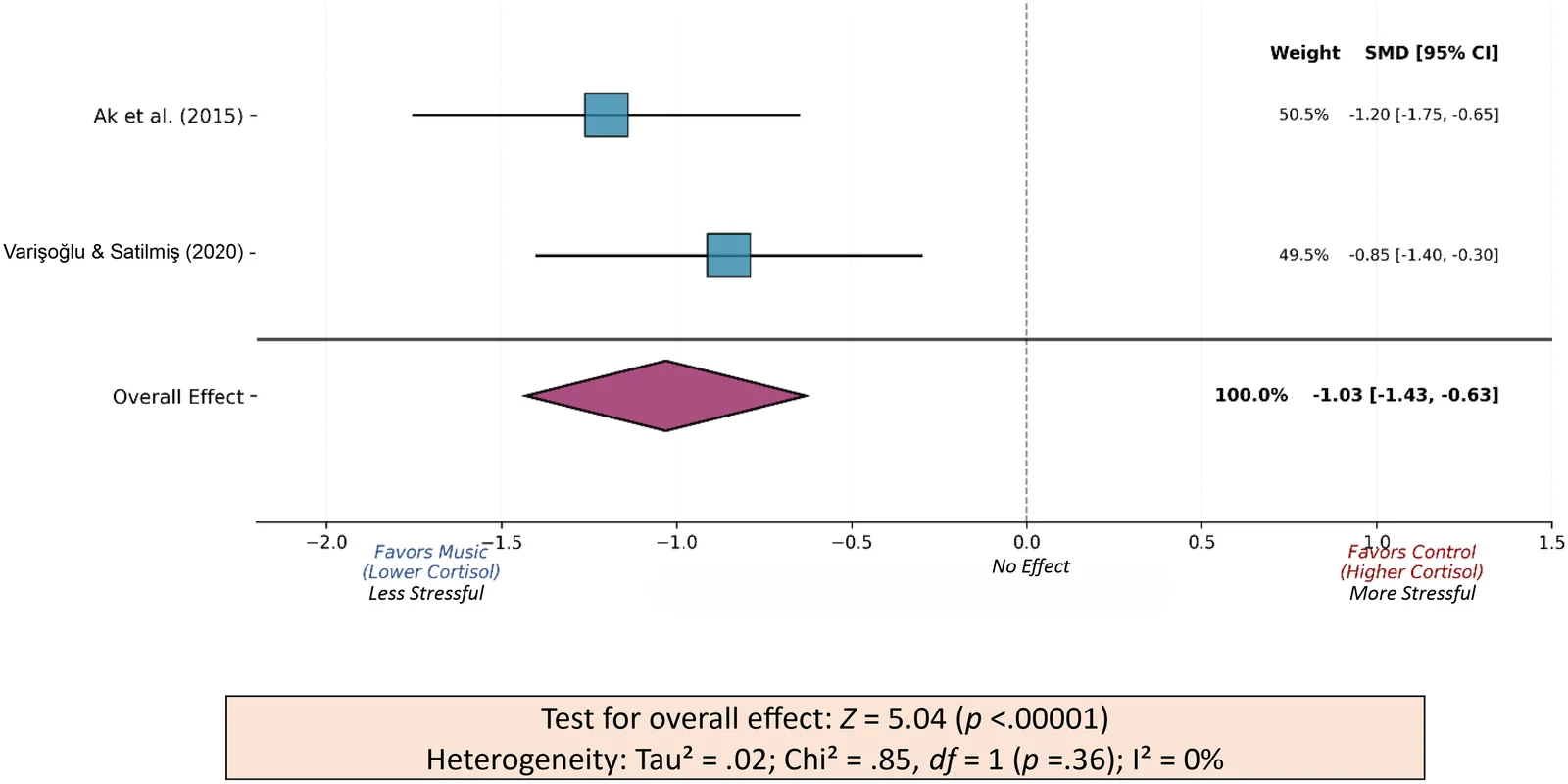
Forest plot – effect of music on salivary cortisol (maternal physiological stress).

Figs. 2, 3 and 4 present Forest Plots that visualize the Standardized Mean Difference (SMD), illustrating the effect of music on breastfeeding outcomes. According to Cohen’s *d* criteria, an SMD of 0.20 indicates a small effect, 0.50 indicates a moderate effect, and 0.80 or greater indicates a large effect size [12]. The data analyzed includes objective measures such as breast milk volume (primary outcome), fat content (secondary outcome), and maternal salivary cortisol stress levels (tertiary outcome).

### Primary outcome: effect of music on breast milk volume

Five of the six studies [15–19] measured breast milk volume. The pooled analysis from the meta-synthesis (see Fig. 2) revealed a consistent, statistically significant positive effect of music on milk production. Immediate effects were observed in studies by Ak et al. (2015) [15] and Kittithanesuan et al. (2017) [17], where music listening led to a significant increase in milk volume per pumping session (*p* < 0.001), suggesting rapid activation of the oxytocin reflex. In contrast, cumulative effects observed by Varişoğlu and Satilmiş (2020) [19] and SefidHaji et al. (2022) [18] showed minimal differences on Day 1, but significantly increases by Day 3 and Day 6, respectively.

The methods for quantifying milk output and accounting for 24-hour natural fluctuations varied across the studies. Specifically, only Keith et al. (2012) [16] adequately captured 24-hour volume variations. In their trial, mothers were encouraged to pump 8 times daily for 14 days using a provided double electric breast pump, with output documented in a standardized pumping record. This frequency is sufficient to cover natural circadian fluctuations in milk production, thereby reducing measurement bias and yielding highly actionable, objective data [24].

In contrast, other included studies lacked comprehensive 24-hour monitoring. Ak et al. (2015) [15] and SefidHaji et al. (2022) [18] measured expressed milk in millilitres (ml) using graduated collecting bottles under researcher supervision. However, measurements were taken infrequently, only once [18] to twice [15, 19] per day, without specifying the overarching pumping schedules for either group. The failure to account for 24-hour variation introduces systematic error, as it remains unclear whether there was a uniform pumping schedule or if mothers had recently emptied their breasts prior to the documented sessions. Similarly, Kittithanesuan et al. (2017) [17] assessed milk production immediately following vaginal delivery (at the first and second hour) by observing the number of drops and flow. While visual cues serve as useful qualitative markers, they lack precision and do not perfectly correlate with the actual volume of milk removed [25], introducing a high risk of measurement bias.

Furthermore, a lack of standardization in the equipment used poses a threat to data reliability. Varişoğlu & Satilmiş (2020) [19] instructed mothers to record production in a daily follow-up form but did not detail the specific measurement tools utilized, potentially compromising data validity [21]. While it is generally assumed that standard electric breast pumps feature graduated containers, and all aforementioned studies provided equipment training, only one study [15] explicitly specified the brand of the breast pump. As different brands and container calibrations can yield slight volumetric discrepancies [26], the lack of equipment standardization across participants and trials warrant future researches to prioritize standardized, 24-hour volumetric tracking using uniform, calibrated equipment to accurately define the clinical magnitude of music interventions on total daily milk yield. 

The Forest Plot in Fig. 2 synthesizes data from the five studies that measured breast milk volume. Vianna et al. (2011) [20] was excluded from the meta-analysis as it did not report breast milk volume as the primary outcome. The horizontal lines represent the 95% Confidence Interval (CI) for each study, while the square indicates the study’s weight (sample size). The center line (0) represents no difference between groups. A square to the right of the center line (> 0) indicates a positive effect favouring music (increased milk volume), while a square to the left (< 0) indicates an effect favouring the control group.

The diamond (♦) at the bottom of the plot represents the pooled effect. It lies to the right of the center line, indicating a statistically significant positive effect, which means that music consistently increases milk volume across all study populations. This conclusion is confirmed by the Z score of 5.41 (*p* < 0.00001), which is statistically significant.

The I^2^ of 4% initially suggests that despite the variation in study locations (Turkey, USA, Iran, Thailand, India), the statistical heterogeneity of the differences is low. However, this metric must be interpreted with caution. With a small number of included studies (*k* = 5), the I^2^ statistic inherently lacks the statistical power to detect true clinical heterogeneity [14]. Although there are potential variations in effect sizes based on infant gestational age (preterm versus term), the data suggests that music exerts a positive effect on milk volume across both populations.

Besides, to account for the use of different measurement tools across the included studies, the Standardized Mean Difference (SMD) was utilized to convert the construct of breast milk volume into a uniform scale, facilitating an overall meta-analytic summary [12]. As described in the primary outcome analysis, the statistical effect size of increased milk volume varied across individual studies. Ak et al. (2015) [15] demonstrated a large effect size (SMD = 1.24). Two studies exhibited moderate effect sizes, with SMDs of 0.56 [16] and 0.62 [19], respectively. Conversely, the statistical effect sizes for SefidHaji et al. (2022) [18] (SMD = 0.48) and Kittithanesuan et al. (2017) [17] (SMD = 0.35) were small.

Overall, as shown in Fig. 2, the intervention groups consistently outperformed the control groups with a pooled SMD of 0.65. While this represents a statistically moderate effect size across the synthesized data, the translation of this finding into a clinical magnitude must be interpreted with caution due to underlying variations in measurement validity, pump standardization, and the frequency of volume tracking across the included trials.

### Long-term outcomes: breastfeeding maintenance

Vianna et al. (2011) [20] assessed long-term breastfeeding outcomes and found that music therapy significantly increased exclusive breastfeeding rates at 60 days post-discharge (*p* = 0.03). This suggests that the immediate benefits of music, such as increased milk volume and relaxation, may translate into sustained breastfeeding success.

### Secondary outcome: breast milk composition

Two studies [16, 18] assessed the impact of music on breast milk composition. Both found significantly higher fat content in the milk of mothers exposed to music. Keith et al. (2012) [16] reported higher caloric density, while SefidHaji et al. (2022) [18] observed elevated triglyceride levels (210.72 versus 177.84 mg/dL) and higher albumin concentrations in the music group. Research [27] indicates that the degree of breast fullness at the beginning of a pumping session is the primary determinant of fat concentration in the expressed milk. Specifically, there is an inverse relationship where a fuller breast results in lower overall fat concentration, while a more depleted breast yields milk with a higher fat content [27].

From Fig. 3, the overall effect of music on breast milk composition, particularly fat content, is statistically significant (*Z* = 5.14, *p* < 0.00001). The pooled effect size (diamond ♦) is positioned well to the right of the zero vertical line (SMD = 0.63), indicating a moderate clinical effect. This suggests that mothers who listened to music produced breast milk with significantly higher fat content compared to those in the control group.

To address maternal physiological variance and prevent unit-of-analysis errors within the individual studies, the included trials utilized relevant statistical modeling. Keith et al. (2012) [16] employed a repeated-measures ANOVA with a Bonferroni correction across a 14-day period, while SefidHaji et al. (2022) [18] utilized an ANCOVA adjusted for baseline lipid profiles. It is important to clarify that these statistical models represent within-study methodological choices to handle multiple measurements and are distinct from the meta-analytic unit-of-analysis decision. These within-study approaches may have strengthened the statistical validity of the reported increases in milk fat.

The consistency between the two studies [16, 18] is excellent, with an I² value of 0%, indicating no observed heterogeneity. Despite being conducted in different decades (2012 versus 2022) and countries (USA versus Iran), the biological impact of music on milk composition remains consistent.

### Tertiary outcomes: maternal stress and psychological impact

Two studies [15, 19] evaluated maternal stress by measuring salivary cortisol concentrations. Varişoğlu and Satilmiş (2020) [19] reported a significant reduction in cortisol levels (*p* < 0.05) in the music group. This reduction in cortisol suggests that music induces a uniform physiological downregulation of the sympathetic nervous system, which may create a conducive biological state for oxytocin release, in which this proposed impression ought to be further researched for.

A meta-analysis of two studies [15, 19] (see Fig. 4) examining maternal stress through salivary cortisol levels demonstrates a large, statistically significant pooled effect in favor of music intervention (SMD = -1.03, *p* < 0.00001). The effect size is illustrated by the combined effect (diamond ♦), which shifts to the left, indicating a strong reduction in cortisol levels. This is further confirmed by the *Z* score for the overall effect (5.04), which is statistically significant (*p* < 0.00001).

Both studies assessed the same stress hormone, with low heterogeneity (I² = 0%), reporting high consistency in the physiological response across different populations (India versus Turkey). This suggests that music can effectively reduce stress response that may inhibit oxytocin release.

### GRADE assessment

Grading of Recommendations Assessment, Development and Evaluation (GRADE) assessment was conducted to rate the certainty of evidence and strength of this systematic review [28]. Since all six included studies were Randomized Controlled Trials (RCTs), the initial certainty of evidence for all outcomes started at “High.”

### Downgrading

The evidence for all outcomes was downgraded by one level due to risk of bias. As is inherent in non-pharmacological trials, it was impossible to blind participants or personnel to the music intervention across all six studies [15–20]. This lack of blinding introduces potential performance bias, particularly for subjective tertiary outcomes.

No downgrades were applied for inconsistency or indirectness. The statistical heterogeneity was remarkably low across pooled outcomes (I^2^ = 0% to 4%), and all studies directly addressed the target populations, interventions, and outcomes defined in this review’s PICO framework.

The evidence for breast milk volume was downgraded by an additional level due to imprecision and measurement bias. As previously noted, the methodologies for quantifying milk yield, such as the lack of 24-hour continuous tracking and the use of non-standardized pumping equipment, varied significantly across the 5 trials [15–19]. Consequently, the overall certainty of evidence for music increasing breast milk volume is rated as “Low”.

Meanwhile, for outcomes utilizing objective laboratory analyses, specifically breast milk composition (fat content) [16, 18] and maternal stress (salivary cortisol) [15, 19], the evidence was not downgraded for measurement bias. Nevertheless, due to the relatively small pooled sample sizes (fewer than 200 participants per outcome), the certainty of evidence for these outcomes is rated as “Moderate”.

Finally, regarding publication bias, while all six trials demonstrated positive directional outcomes favouring the intervention, raising the possibility of asymmetric reporting, the risk was deemed “suspected” rather than “strongly suspected” [29]. Therefore, it did not meet the strict GRADE threshold for an additional one-level downgrade [29]. Furthermore, the small number of included trials (*k* = 6) limits the reliable statistical evaluation of publication bias, as standard tests for funnel plot asymmetry require a minimum of 10 studies to adequately distinguish chance from real asymmetry [12]. Thus, this potential risk is narratively acknowledged as a necessary caution in the “Limitations” section rather than applied as a formal downgrading to the certainty ratings.

### Upgrading

According to GRADE guidelines, evidence can be upgraded based on three specific criteria: a large magnitude of effect, a dose-response gradient, or if all plausible confounding would reduce a demonstrated effect [28]. In this review, no upgrading criteria were met. While the meta-analysis demonstrated a statistically significant positive effect on breast milk volume, the pooled effect size (SMD = 0.65) represents a moderate, rather than a large, clinical magnitude. Furthermore, while a study [19] suggested a cumulative benefit of music over multiple days, a formal, standardized dose-response gradient was not established across the included trials [15–20]. Consequently, the certainty of the evidence was not upgraded.

**Table 3:** GRADE assessment

Outcome	Level of Certainty^a^	Authors
**Primary outcomes** Objective breastfeeding metrics • Breast milk volume • Breastfeeding duration	Low Low	Ak et al. (2015) [15] Keith et al. (2012) [16] Kittithanesuan et al. (2017) [17] SefidHaji et al. (2022) [18] Varişoğlu and Satilmiş (2020) [19] Vianna et al. (2011) [20]
**Secondary outcome** Breast milk composition (fat and protein)	Moderate	Keith et al. (2012) [16] SefidHaji et al. (2022) [18]
**Tertiary outcomes** Objective • Maternal stress marker - salivary cortisol levels Subjective • Psychological status - anxiety, perceived stress via PSS-14	Moderate Low	Ak et al. (2015) [15] Varişoğlu and Satilmiş (2020) [19] Ak et al. (2015) [15] Varişoğlu and Satilmiş (2020) [19]

^a^ Publication bias was suspected due to consistent positive directional outcomes across all included trials; however, the evidence was explicitly not downgraded for this domain. The risk did not meet the strict GRADE threshold for “strongly suspected” [28], and formal statistical testing for publication bias, such as funnel plot asymmetry, was precluded by the small number of studies (*k* < 10) [12]

In summary, based on the GRADE framework, the final certainty of evidence for the evaluated outcomes ranges from “Moderate to Low” as shown in Table 3. The initial “High” rating for all Randomized Controlled Trials (RCTs) was downgraded primarily due to inherent risks of bias (lack of blinding) and methodological imprecisions in measurement. While the meta-analysis indicates statistically significant benefits of music on total daily milk volume, the GRADE assessment highlights that the true clinical magnitude of these effects is uncertain. Future rigorously designed RCTs with standardized 24-hour volumetric tracking and larger sample sizes are required to elevate the certainty of this evidence.

## Discussion

This systematic review and meta-analysis suggests that music intervention can be a beneficial, non-pharmacological adjunct to standard lactation care. Evidence from diverse clinical settings, including the NICU and routine postpartum wards, indicates that music appears to support increases in breast milk volume and lipid content, while simultaneously reducing maternal stress.

The data in this review suggests that auditory stimulation positively impacts breast milk volume and overall lactation performance, with evidence indicating that these benefits may be dose-dependent and cumulative. For instance, while Kittithanesuan et al. (2017) [17] observed immediate improvements in milk ejection time following a single intervention, Varişoğlu and Satilmiş (2020) [19] found that significant increases in milk volume emerged only after consistent music intervention over several days. This immediate and cumulative physiological support may contribute to long-term behavioural success. Experiencing a faster and more productive let-down reflex might better equip mothers to persist through early breastfeeding challenges. This aligns with findings indicating that mothers exposed to music therapy demonstrated significantly higher breastfeeding maintenance rates 60 days post-discharge (Vianna et al., 2011) [20].

Beyond milk volume, recent studies add a critical dimension by highlighting music’s positive impact on breast milk composition, specifically fat content [16, 18]. This increase in lipid concentration is likely mediated by the neurological regulation of the milk ejection reflex (MER). While all breast milk is nutritious, lipid concentration naturally rises as the breast is progressively drained, a process requiring multiple MERs per session [25, 27]. However, when stress blunts the myoepithelial contractions in the breast, fat globules remain trapped in the alveolar walls [4]. By potentially facilitating a more productive let-down reflex, music may aid in a more complete evacuation of the breast, theoretically dislodging energy-dense breast milk and contributing to the increased overall lipid concentration observed in the expressed milk.

The improvements observed in the primary and secondary outcomes are hypothesized to be associated with music’s ability to reduce maternal stress and anxiety. Stressors associated with the NICU environment or physical recovery from childbirth are known to trigger the maternal hypothalamic-pituitary-adrenal (HPA) axis [18, 19]. The resulting elevation in cortisol and catecholamines can constrict capillaries around the mammary alveoli and suppress oxytocin release [4]. Auditory stimulation appears to lessen this neuroendocrine barrier. By significantly reducing maternal stress and salivary cortisol levels [7, 19], music may promote a shift from sympathetic arousal (fight or flight) to parasympathetic dominance (rest and digest). Psychologically, by alleviating anxiety and performance pressure, music may help preserve maternal self-efficacy and encourage mothers to associate breastfeeding with relaxation rather than stress [24].

Nonetheless, it is critical to acknowledge that the overarching causal pathway, specifically the assumption that music directly resolves the cortisol-oxytocin conflict, remains a theoretical inference. None of the included studies simultaneously measured cortisol and oxytocin, nor did they directly track the physical mechanics of breast milk ejection. Consequently, the simultaneous quantification of these paired biomarkers during auditory interventions remains a crucial priority for future research to definitively confirm this proposed neuroendocrine pathway.

Despite the low statistical heterogeneity (I^2^) observed in the meta-analyses, there is substantial clinical and methodological heterogeneity across the included literature that warrants discussion. Clinically, the interventions varied significantly in both modality and dosage. Auditory stimuli ranged from live classical Indian raga and recorded Western classical music to cultural pop songs and lullabies. Furthermore, the duration and frequency of these interventions ranged from a single 15-minute session immediately postpartum to 60-minute sessions spanning up to 14 days. The study populations and clinical settings also represent distinct clinical paradigms. Mothers of preterm infants and healthy term infants generally experience differently. Methodologically, the tracking of outcomes varied from single-day, immediate post-intervention volume assessments to cumulative tracking over several days. While auditory stimulation consistently yielded positive directional outcomes across these diverse parameters, this clinical and methodological heterogeneity means that a standardized, optimal dose or genre of music therapy for lactation support has yet to be definitively established.

### Clinical implications and recommendations

Based on the synthesized evidence in this review, healthcare providers might consider exploring music integration alongside standard pumping practices. For mothers of preterm infants, pumping is often a mechanical and isolating task. Hospitals could suggest listening to 15–30 min of soothing music during pumping sessions by drawing on protocols utilized by Varişoğlu and Satilmiş (2020) [19] and Ak et al. (2015) [15]. This low-cost intervention can be easily incorporated into existing care via personal devices. Additionally, clinicians might discuss a multi-sensory approach to help support the let-down reflex. SefidHaji et al. (2022) [18] observed that combining music with visual stimuli (such as viewing a photo of the infant) was associated with higher milk volumes and protein levels. This approach mimics the natural breastfeeding experience, where the mother sees, touches, and hears her baby, which may theoretically help facilitate a more favorable hormonal environment. In addition, a simultaneous cortisol-oxytocin measurement could be a future research priority to better understand the proposed neuroendocrine pathway.

For routine deliveries, findings from Kittithanesuan et al. (2017) [17] suggest that the use of music within the first few hours postpartum may help facilitate the initial latch, which could be particularly beneficial for primiparous mothers or those recovering from challenging deliveries. In NICUs, which are often noisy environments, hospitals might consider providing noise-canceling headphones with music. This could help create a more controlled auditory environment, potentially assisting mothers in managing clinical distractions and focusing on bonding with their infants.

### Limitations

Although this review was not registered with the International Prospective Register of Systematic Reviews (PROSPERO), this review is part of the university’s registered project with an identification number of F05/CDRG/1829/2019.

A primary methodological limitation of this review involves the consistency and validity of breast milk volume measurements. Since the methods for quantifying milk output and accounting for natural 24-hour fluctuations varied across the included trials, the overall clinical effect sizes must be interpreted with caution. Beyond these measurement disparities, there is clinical heterogeneity regarding the infants’ gestational ages and the specific types of music utilized. Furthermore, the inherent inability to blind participants to auditory interventions introduces a risk of performance bias, particularly for subjective outcomes such as anxiety. To address these variables, future research could explore standardizing acoustic parameters and volumetric measurement to better identify optimal conditions for lactation support.

Furthermore, a fundamental limitation of the current evidence base is the reliance on theoretical neuroendocrine pathways. As oxytocin and cortisol dynamics were not simultaneously measured in real-time alongside milk expression in the included trials, the assumption that music directly resolves the cortisol-oxytocin conflict remains an inference. This limits the ability to draw definitive biological conclusions regarding the exact mechanisms driving the observed improvements in milk volume and composition.

Another important limitation of this review is the suspected risk of publication bias. It is notable that all six included trials reported positive directional outcomes favouring the music intervention. While this consistency is encouraging, there is a distinct possibility of unpublished null findings, where studies demonstrating no significant difference between groups fail to reach publication. Additionally, the small total number of included trials (*k* = 6) precluded the use of formal statistical assessments for small-study effects, such as Egger’s test or funnel plot asymmetry, which require a minimum of 10 studies to yield reliable results [12]. Consequently, this suspected risk of publication bias could not be statistically quantified. This potential overrepresentation of positive results suggests that the current literature may overstate the true clinical efficacy of the intervention, further necessitating a cautious interpretation of the pooled effect sizes and reinforcing the ‘Moderate to Low’ certainty of evidence yielded by the GRADE assessment.

Finally, there is an additional meta-analytic limitation in the handling of data across multiple time points. Since several included trials reported milk volume longitudinally, the meta-analysis strictly utilized the final post-intervention measurements to avoid unit-of-analysis errors. While this is a methodologically sound approach for statistical pooling, it means the synthesized effect sizes capture a specific endpoint rather than the full trajectory of day-to-day milk production changes.

### Novelty and uniqueness of the study

This review uniquely synthesizes the physiological and psychological impacts of music on breastfeeding across diverse settings and populations, including both term and preterm infants. By evaluating multiple outcomes, such as milk volume, composition, cortisol stress level, and long-term maintenance, it explores how music may facilitate breastfeeding. Ultimately, this review offers valuable insights to inform clinical practice and future research.

## Conclusion

The findings from six randomized controlled trials suggest that music intervention may positively influence lactation performance. Current evidence indicates that music is associated with increased lipid content in breast milk, as well as lowered cortisol levels and reduced anxiety and stress. Although only one study examined long-term outcomes, it suggests that early music intervention may be associated with higher breastfeeding maintenance rates post-discharge. Given its low cost, non-invasive nature, and potential absence of adverse effects, music could serve as a valuable adjunct to standard lactation support. Clinicians may consider encouraging mothers to listen to soothing or preferred music for at least 15 min during breast milk expression to potentially promote relaxation and support milk yield. However, further rigorously designed trials are necessary to standardize optimal protocols and definitively confirm the clinical magnitude of these benefits.

## Supplementary Information

Below is the link to the electronic supplementary material.

Supplementary Material 1

## Data Availability

Link where all 6 reviewed papers are available: https://drive.google.com/drive/folders/1JEOzaVjXSvpzCZ3tN3BVllq5MBBVLJM4?usp=sharing. Alternatively, the links to the websites have been checked, amended, and rechecked: Ak, J., Lakshmanagowda, P. B., Pradeep, G. C. M. & Goturu, J. (2015). Impact of music therapy on breast milk secretion in mothers of premature newborns. Journal of clinical and diagnostic research, 9(4), CC04–CC6. https://doi.org/10.7860/JCDR/2015/11642.5776. Keith, D. R., Weaver, B. S., & Vogel, R. L. (2012). The effect of music-based listening interventions on the volume, fat content, and caloric content of breast milk produced by mothers of premature and critically ill infants. Advances in Neonatal Care, 12(2), 112–119. https://www.researchgate.net/publication/223982580. Kittithanesuan, Y., Chiarakul, S., Kaewkungwal, J. and Poovorawan, Y. (2017). Effect of music on immediately postpartum lactation by term mothers after giving birth: A randomized controlled trial. Journal of the Medical Association of Thailand, 100(8), 834–842. http://jmatonline.com/view.php?id=1777. SefidHaji, S., Aziznejadroshan, P., Mojaveri, M. H., Nikbakht, H. A., Qujeq, D., & Amiri, S. R. J. (2022). Effect of lullaby on volume, fat, total protein and albumin concentration of breast milk in premature infants' mothers admitted to NICU: a randomized controlled trial. International breastfeeding journal, 17(1), 71. https://doi.org/10.1186/s13006-022-00511-7. Varişoğlu, Y., & Satilmiş, I. G. (2020). The Effects of Listening to Music on Breast Milk Production by Mothers of Premature Newborns in the Neonatal Intensive Care Unit: A Randomized Controlled Study. Breastfeeding Medicine: The Official Journal of the Academy of Breastfeeding Medicine, 15(7), 465–470. https://doi.org/10.1089/bfm.2020.0027. Vianna, M. N., Barbosa, A. P., Carvalhaes, A. S., & Cunha, A. J. (2011). Music therapy may increase breastfeeding rates among mothers of premature newborns: a randomized controlled trial. Jornal de Pediatria, 87(3), 206–212. https://doi.org/10.15845/voices.v12i3.678.

## Abbreviations

CI: Confidence Interval
HPA: Hypothalamic-Pituitary-Adrenal (axis)
MER: Milk Ejection Reflex
NICU: Neonatal Intensive Care Unit
OR: Odds Ratio
PRISMA: Preferred Reporting Items for Systematic Reviews and Meta-Analyses
PSS: Perceived Stress Scale
RCT: Randomized Controlled Trial
RevMan: Review Manager (Software)
RoB 2: Risk of Bias 2 (Cochrane tool)
SMD: Standardized Mean Difference
STAI: State-Trait Anxiety Inventory
GRADE: Grading of Recommendations Assessment, Development and Evaluation
PROSPERO: International Prospective Register of Systematic Reviews
